# A Comparison of Forensic Age Prediction Models Using Data From Four DNA Methylation Technologies

**DOI:** 10.3389/fgene.2020.00932

**Published:** 2020-08-19

**Authors:** A. Freire-Aradas, E. Pośpiech, A. Aliferi, L. Girón-Santamaría, A. Mosquera-Miguel, A. Pisarek, A. Ambroa-Conde, C. Phillips, M. A. Casares de Cal, A. Gómez-Tato, M. Spólnicka, A. Woźniak, J. Álvarez-Dios, D. Ballard, D. Syndercombe Court, W. Branicki, Ángel Carracedo, M. V. Lareu

**Affiliations:** ^1^Forensic Genetics Unit, Institute of Forensic Sciences, University of Santiago de Compostela, Galicia, Spain; ^2^Malopolska Centre of Biotechnology, Jagiellonian University, Kraków, Poland; ^3^King’s Forensics, Department of Analytical, Environmental and Forensic Sciences, Faculty of Life Sciences and Medicine, King’s College London, London, United Kingdom; ^4^Faculty of Mathematics, University of Santiago de Compostela, Galicia, Spain; ^5^Central Forensic Laboratory of the Police, Warsaw, Poland; ^6^Fundación Pública Galega de Medicina Xenómica – CIBERER-IDIS, Santiago de Compostela, Spain

**Keywords:** epigenetics, DNA methylation, age estimation, EpiTYPER^®^, pyrosequencing, MiSeq, SNaPshotcpsdummy^TM^

## Abstract

Individual age estimation can be applied to criminal, legal, and anthropological investigations. DNA methylation has been established as the biomarker of choice for age prediction, since it was observed that specific CpG positions in the genome show systematic changes during an individual’s lifetime, with progressive increases or decreases in methylation levels. Subsequently, several forensic age prediction models have been reported, providing average age prediction error ranges of ±3–4 years, using a broad spectrum of technologies and underlying statistical analyses. DNA methylation assessment is not categorical but quantitative. Therefore, the detection platform used plays a pivotal role, since quantitative and semi-quantitative technologies could potentially result in differences in detected DNA methylation levels. In the present study, we analyzed as a shared sample pool, 84 blood-based DNA controls ranging from 18 to 99 years old using four different technologies: EpiTYPER^®^, pyrosequencing, MiSeq, and SNaPshot^TM^. The DNA methylation levels detected for CpG sites from *ELOVL2*, *FHL2*, and *MIR29B2* with each system were compared. A restricted three CpG-site age prediction model was rebuilt for each system, as well as for a combination of technologies, based on previous training datasets, and age predictions were calculated accordingly for all the samples detected with the previous technologies. While the DNA methylation patterns and subsequent age predictions from EpiTYPER^®^, pyrosequencing, and MiSeq systems are largely comparable for the CpG sites studied, SNaPshot^TM^ gives bigger differences reflected in higher predictive errors. However, these differences can be reduced by applying a z-score data transformation.

## Introduction

DNA methylation is the most widely studied epigenetic mark in the human genome (Jones, 2012). Methylation, which is the incorporation of a methyl group at cytosine-guanine dinucleotide motifs, has been shown to be highly correlated with the human aging process (Bocklandt et al., 2011; Hannum et al., 2013; Horvath, 2013; Johansson et al., 2013), and as a consequence, is currently considered the most accurate age prediction biomarker. The estimation of an individual’s epigenetic age in this way is useful in several areas. From a forensic point of view, the ability to accurately predict the chronological age of the donor of a biological sample can provide relevant information in order to guide police investigation in cases where no suspects or matches in the DNA database are found (Freire-Aradas et al., 2017). From a clinical point of view, biological age estimation may help to determine the life expectancy of the individual (Horvath and Raj, 2018). In order to infer the chronological age, several age prediction models have been developed based on data generated using different DNA methylation technologies, including EpiTYPER^®^ (Xu et al., 2015; Freire-Aradas et al., 2016; Zubakov et al., 2016), pyrosequencing (Weidner et al., 2014; Bekaert et al., 2015; Zbieć-Piekarska et al., 2015), massively parallel sequencing (MPS) (Naue et al., 2017; Vidaki et al., 2017; Aliferi et al., 2018) and SNaPshot^TM^ (Lee et al., 2015; Hong et al., 2017; Jung et al., 2019) systems. As DNA methylation is quantitative in nature, potential differences in DNA methylation levels can be detected by each technology. For this reason, systematic comparisons of methylation detection technologies using a common set of controls become necessary.

A shared characteristic of these aforementioned DNA methylation technologies is their reliance on bisulfite conversion of the analyzed samples. Bisulfite conversion is a pretreatment of the genomic DNA that coverts all the unmethylated cytosines to uracil, which after PCR, are replaced with thymine; while the methylated CpGs remain unaltered; converting a methylation difference into a sequence difference (Frommer et al., 1992; Clark et al., 1994). Two main limitations are related to the bisulfite conversion process. First, it degrades the DNA and consequently, a larger amount of genomic DNA than usual must be used. Second, it reduces DNA sequence variability, diminishing the multiplexing capacity of the technologies used. In spite of these constraints, the high quality of the corresponding DNA methylation measurements favors their use in current forensic applications.

EpiTYPER^®^ detects and quantifies DNA methylation based on MALDI-TOF mass spectrometry (Ehrich et al., 2006). EpiTYPER^®^ analyses the cleaved fragments with varied molecular weights depending on the methylation status around each fragment, which are measured and the corresponding DNA methylation levels ascertained. Although single-nucleotide CpG positions are measured, some CpGs very close to each other are detected as a block providing an average methylation value for a set of two to three CpG sites. Pyrosequencing is considered the gold standard method for measuring targeted DNA methylation (Lehmann and Tost, 2015). It is an accurate quantitative sequencing-by-synthesis method based on luminescence, following an enzymatic cascade that uses the production of light after pyrophosphate release when a nucleotide is incorporated onto a growing DNA strand. Despite its high accuracy, it is difficult to analyze multiple markers simultaneously, hindering its use in forensic casework where the quality/quantity of DNA samples is usually restricted. MPS (also called next generation sequencing or NGS) is a high-throughput technology that sequences multiple target-specific regions in parallel and quantitatively detects DNA methylation levels by the ratio of sequence read coverage amongst targets (Richards et al., 2018). For forensic casework, two main companies provide the equipment necessary to run DNA methylation analysis with MPS technology: Illumina using the MiSeq system, and Thermo Fisher using the Ion S5 detector. MiSeq technology uses sequencing-by-synthesis where a fluorescently labeled reversible terminator is imaged as each dNTP is added, and then cleaved to allow incorporation of the next base. Ion Torrent technology is based on semiconductor sequencing, i.e., each time a nucleotide is incorporated in the growing DNA strand, a proton is released and the consequent variation in pH is measured as a change in electrical conductivity. MPS appears to be a highly accurate technology for DNA methylation analysis while allowing for multiplexing. Nevertheless, the high cost associated with both the equipment and reagents is a constraining factor for some forensic laboratories that require a more cost-effective technology such as single base extension (SBE) that can be easily incorporated into well-established capillary electrophoresis systems. SBE (also called minisequencing or SNaPshot^TM^) is a semi-quantitative technology based on fluorescence (Fondevila et al., 2017). It consists of the annealing of an unlabeled oligonucleotide that matches the sequence immediately adjacent to the target nucleotide site. The subsequent incorporation of a single complementary fluorescently labeled terminator ddNTP produces a sequence strand extended by one nucleotide. While the multiplex capacity of this method is an advantage, the different fluorescence intensities between each of the dyes linked to the ddNTPs used, can potentially bias the methylation values detected.

Up until now, age prediction models have been developed based on data collected using one technology, i.e., if an age prediction model is built based on pyrosequencing data, the subsequent test samples are also analyzed by pyrosequencing, and so on, since some loss of accuracy was previously reported for inter-technology data exchange (Vidaki et al., 2017; Aliferi et al., 2018). This represents a constraint, since each technology requires the re-building of the prediction model with new age reference sample sets. We aimed to compare DNA methylation data from different technologies in order to explore if platform-independent models might be useful for forensic age prediction. The study of Hong et al. (2019) has already introduced this concept by developing a platform-independent model for MPS and SNaPshot^TM^ in saliva samples. In the present study, we cover a further inter-technology comparison for DNA methylation based on MPS and SNaPshot^TM^ technologies, but adding methods based on EpiTYPER^®^ and pyrosequencing. A total of 84 common control DNAs from blood between 18 and 99 years old were analyzed using the four different technologies for three CpG sites in *ELOVL2*, *FHL2*, and *MIR29B2* genes. The corresponding DNA methylation levels were compared, and several age prediction models were subsequently tested in the common samples detected with either the same or different technologies to the system used to build the training set.

## Materials and Methods

### DNA Samples and DNA Methylation Data

A total of 84 blood sample-derived DNA extracts were obtained from healthy European volunteers ranging in age from 18 to 99 years. The samples were used as the testing set (referenced as common controls). All samples were obtained from the ‘Carlos III’ Spanish National DNA Bank, University of Salamanca, and ethical approval was granted from the ethics committee of investigation in Galicia, Spain (CAEI: 2013/543). All DNA samples were quantified by Qubit^®^ dsDNA High Sensitivity (HS) Assay Kit (Thermo Fisher) and subsequently normalized to 10 ng/μL. Additionally, DNA methylation data for a total of 1130 European blood samples ranging in age from 2 to 104 years were selected from previous studies that used EpiTYPER^®^ (*N* = 725) (Freire-Aradas et al., 2016), pyrosequencing (*N* = 293) (Zbieć-Piekarska et al., 2015), and MiSeq (*N* = 112) (Aliferi et al., 2018) for building the training sets. Moreover, a total of 105 European blood samples ranging in age from 18 to 75 years were analyzed using SNaPshot^TM^ in order to build the corresponding training set.

### CpG Sites Selection and DNA Methylation Detection

A total of three age-correlated genes were used for comparative purposes: *ELOVL2*, *FHL2*, and *MIR29B2* – loci that have been commonly included in age prediction models analyzing blood-based DNA samples (Garagnani et al., 2012; Bekaert et al., 2015; Zbieć-Piekarska et al., 2015; Freire-Aradas et al., 2016; Park et al., 2016; Zubakov et al., 2016; Hong et al., 2019; Jung et al., 2019). In the present study, these three genes were analyzed by three independent laboratories using four DNA methylation technologies: EpiTYPER^®^, pyrosequencing, MiSeq, and SNaPshot^TM^. Table 1 describes the overlap between CpG sites and DNA methylation technologies used in this study. All CpGs are single CpG sites, with the exception of *MIR29B2_C1* that consisted of a cluster of three CpG sites, since EpiTYPER^®^ could not give individual results for each site. Therefore, an average of the three corresponding CpG sites was used when comparing the corresponding DNA methylation values with pyrosequencing or MiSeq for this case. Regarding the overlap in Table 1; *ELOVL2*, *FHL2*, and *MIR29B2_C1* were used for comparing EpiTYPER^®^, pyrosequencing, and MiSeq; whereas SNaPshot^TM^ comparisons were made in a separate analysis. In this case, *ELOVL2*, *FHL2*, and *MIR29B2_C2* were used for comparing EpiTYPER^®^ and MiSeq systems with the SNaPshot^TM^ system. Analyses were extended to more than one CpG per gene in the case of *MIR29B2* due to a lack of complete overlap between technologies. *ELOVL2* and *FHL2* were represented by the same CpG site in all analyses. All four DNA methylation technologies require a pretreatment with sodium bisulfite. Three bisulfite kits were used according to the methylation detection technology that will be described below. Supplementary Table S1 summarizes additional variable factors between technologies.

**TABLE 1:** Overlap between CpG sites from the target genes *ELOVL2*, *FHL2*, and *MIR29B2* in the four evaluated DNA methylation technologies: (A) EpiTYPER^®^, (B) Pyrosequencing, (C) MiSeq, and (D) SNaPshot^TM^.

**Gene**	**CpG_ID**	**GRCh38 position**	**(A) EpiT**	**(B) Pyros**	**(C) MiSeq**	**(D) SNaP**
*ELOVL2*	cg21572722	chr6:11044661	√	√	√	√
*FHL2*	cg06639320	chr2:105399282	√	√	√	√
*MIR29B2_C1*	–/cg10501210/-	chr1:207823672/75/81	√	√	√	
*MIR29B2_C2*	–	chr1:207823715	√		√	√

*All CpGs are single sites, except *MIR29B2_C1* that is a cluster of three CpG sites. The corresponding DNA methylation values for *MIR29B2_C1* were calculated as the average of the three CpG sites for each technology.*

### Agena Bioscience EpiTYPER^®^ DNA Methylation Analysis

The Agena Bioscience EpiTYPER^®^ system (San Diego, CA, United States) used PCR amplicons of 362 base pairs (bp) for *ELOVL2*, 191 bp for *FHL2* and 344 bp for *MIR29B2*. Samples analyzed using EpiTYPER^®^ were bisulfite converted using the EZ DNA Methylation^TM^ Kit (Zymo Research) using 300 ng of genomic DNA. A detailed description of the EpiTYPER^®^ workflow has been previously reported (Freire-Aradas et al., 2016, 2018). Methylation data were obtained using EpiTYPER^®^ software v.1.2.22 (Agena Bioscience).

### Pyrosequencing

Pyrosequencing of the PCR amplicons used for this technology were 308 bp for *ELOVL2*, 167 bp for *FHL2* and 146 bp for *MIR29B2*. Bisulfite conversion was performed using the Qiagen 96-well bisulfite conversion kit (Qiagen, Hilden, Germany) using 1 μg of genomic DNA. Specific procedures for the pyrosequencing workflow were previously outlined (Zbieć-Piekarska et al., 2015). This technology was performed using a PyroMark vacuum prep workstation and a PyroMark Q24 instrument (Qiagen), following the manufacturer’s guidelines. The data were automatically analyzed using PyroMark analysis software (Qiagen, Hilden, Germany).

### The MiSeq System

Massively parallel sequencing-based detection of methylated DNA was performed using the MiSeq system (Illumina). Samples detected using MiSeq were bisulfite converted using the MethylEdge^®^ Bisulfite Conversion System (Promega Corporation, Fitchburg, WI, United States) using 50 ng of genomic DNA. Detailed information regarding the workflow can be found in Aliferi et al. (2018). The amplicon sizes used were 308 bp for *ELOVL2*, 165 bp for *FHL2* and 210 bp for *MIR29B2*. Analysis of the FASTQ files was conducted with the Burrows-Wheeler Aligner, Sequence Alignment/Map and Genome Analysis Toolkit software following guidelines from Aliferi et al. (2018).

### Single Base Extension (SBE)

Single base extension was performed using the SNaPshot^TM^ Multiplex Kit (Thermo Fisher) in replicate analyses. PCR amplicon sizes were 111 bp for *ELOVL2*, 108 bp for *FHL2* and 49 bp for *MIR29B2*. Samples for SNaPshot^TM^ analyses were bisulfite converted using the MethylEdge^®^ Bisulfite Conversion System (Promega Corporation, Fitchburg, WI, United States, assay B) using 100 ng of genomic DNA. Specific multiplex protocol details are summarized in Supplementary Table S2. Methylation values were calculated based on the peak height ratio [methylated signal/(methylated signal + unmethylated signal)] obtained with GeneMapperID v3.2.1 software, measuring RFU values (relative fluorescence units). The average of the DNA methylation values between replicates were used for SNaPshot^TM^ analyses.

### Statistical Analyses

Comparisons of DNA methylation measurement methods were performed using Bland-Altman plots using the *BlandAltmanLeh* R package (Lehnert, 2015). These plots represent the difference between paired measures for the same variable against the corresponding mean (Giavarina, 2015). The upper and lower limits of agreement (LoA) were calculated as the mean of the differences between two measurements ±1.96 times the standard deviation (SD) between them, accordingly, in order to include 95% of the differences within them (Bland and Altman, 1999). A limit of acceptance, or threshold of ±0.098 has been established *a priori* (±1.96.SD, SD = 0.05). Normality was tested using a Shapiro–Wilk normality test. Uniformity, i.e., absence of tendency for DNA methylation differences between methods, was checked using regression analysis (*p*-value for the fitted regression line). Differences in DNA methylation levels between technologies were also explored using analysis of variance (ANOVA).

All age prediction modeling was based on quantile regression (QR) (Freire-Aradas et al., 2016; Smeers et al., 2018). The original datasets for the training sets were composed of a higher number of samples and CpG sites than the data required for the reported analyses; specifically: *N* = 725, 18–104 years, 7 CpGs; *N* = 293, 2–75 years, 5 CpGs and *N* = 112, 11–93 years, 12 CpGs measured by EpiTYPER^®^, pyrosequencing, and MiSeq, respectively (Zbieć-Piekarska et al., 2015; Freire-Aradas et al., 2016; Aliferi et al., 2018). Therefore, model re-building was performed in order to harmonize age distribution and sample size, as well as to cover only the three CpG sites under study. The age range was restricted to 18–75 years old for all the training sets. Age range restriction directly led to *N* = 100 for MiSeq. In the case of EpiTYPER^®^ and pyrosequencing, besides age range restriction, random selection of a maximum of two individuals per year-of-age led to *N* = 116 for EpiTYPER^®^ and *N* = 106 for pyrosequencing. Quantiles 0.1 and 0.9 (q10 and q90) were used for the development of the multivariate QR model using the *quantreg* R package (Koenker et al., 2019). Random cleavage of the input data for the QR model validation was done using the *cvTools* R package (Alfons, 2015). Validation of the QR model was performed using k−fold cross−validation (*k* = 10). The corresponding predictive accuracy was measured with the following performance metrics: median absolute prediction error (MAE); root-mean-square error (RMSE); percent of correct predicted samples with a prediction error of ±5 years (%CP ± 5) and percent of correct predicted samples within the prediction intervals (%CP ± PI). Predicted versus chronological age was plotted using the *ggplot2* R package (Wickham and Chang, 2019). Z-score transformation was performed scaling the DNA methylation levels to the corresponding mean and SD. All calculations were performed using R software v3.4.2.

## Results

### Intra-Technology Variation

Intra-technology variation was assessed analyzing two replicates for the semi-quantitative technology used in the present study, i.e., SNaPshot^TM^. Previous work on EpiTYPER^®^ (Freire-Aradas et al., 2016), pyrosequencing (Zbieć-Piekarska et al., 2015), and MiSeq (Aliferi et al., 2018) showed absence of technical variation for these DNA methylation technologies and therefore, the corresponding common controls were analyzed using a single replicate.

Supplementary Figure S1 depicts the DNA methylation levels against the chronological age for *ELOVL2*, *FHL2*, and *MIR29B2_C2* for both replicates for the 84 common controls. An absence of statistically significant differences between replicates (*p*-value > 0.01) allowed the study to use the average DNA methylation levels for SNaPshot^TM^ analyses.

### Comparison of DNA Methylation Levels for EpiTYPER^®^ vs. Pyrosequencing vs. MiSeq vs. SNaPshot^TM^

**FIGURE 1:**
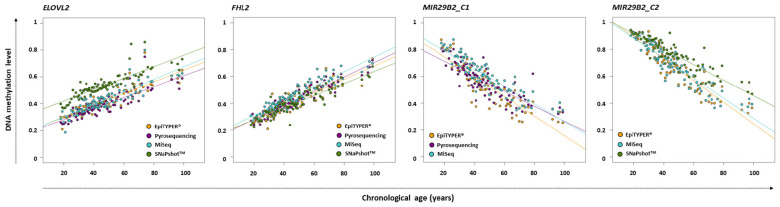
DNA methylation levels compared with chronological age in *ELOVL2*, *FHL2*, *MIR29B2_C1*, and *MIR29B2C_C2* for 84 common controls (18–99 years old) detected using EpiTYPER^®^ (orange), pyrosequencing (dark magenta), MiSeq (light blue), and SNaPshot^TM^ (green).

DNA methylation analysis for *ELOVL2*, *FHL2*, and *MIR29B2* for the 84 common controls was performed using EpiTYPER^®^, pyrosequencing, MiSeq, and SNaPshot^TM^ technologies. The corresponding DNA methylation data (β-values) is shown in Supplementary Table S3. Figure 1 shows the DNA methylation levels against the chronological age for *ELOVL2*, *FHL2*, *MIR29B2_C1*, and *MIR29B2_C2* for the overlapping technologies, while Table 2 describes the corresponding ANOVA test. The major differences were displayed by *ELOVL2* and *MIR29B2_C2* detected using SNaPshot^TM^ technology. Additionally, moderate statistically significant differences were also found for *MIR29B2_C1* comparing EpiTYPER^®^ vs. MiSeq (*p*-value: 0.00303).

**TABLE 2:** ANOVA test for evaluation of the differences between the four DNA methylation technologies studied.

	***p*-value**
***ELOVL2***	
EpiTYPER^®^ vs. pyrosequencing	0.066
EpiTYPER^®^ vs. MiSeq	0.447
Pyrosequencing vs. MiSeq	0.012
EpiTYPER^®^ vs. SNaPshot^TM^	**10^–13^**
Pyrosequencing vs. SNaPshot^TM^	**2 × 10^–16^**
MiSeq vs. SNaPshot^TM^	**6.1 × 10^–11^**
***FHL2***	
EpiTYPER^®^ vs. pyrosequencing	0.636
EpiTYPER^®^ vs. MiSeq	0.0113
Pyrosequencing vs. MiSeq	0.0369
EpiTYPER^®^ vs. SNaPshot^TM^	0.309
Pyrosequencing vs. SNaPshot^TM^	0.128
MiSeq vs. SNaPshot^TM^	**0.000257**
***MIR292B_C1***	
EpiTYPER^®^ vs. pyrosequencing	0.476
EpiTYPER^®^ vs. MiSeq	**0.00303**
Pyrosequencing vs. MiSeq	0.0111
***MIR292B_C2***	
EpiTYPER^®^ vs. MiSeq	0.631
EpiTYPER^®^ vs. SNaPshot^TM^	**7.63 × 10^–5^**
MiSeq vs. SNaPshot^TM^	**0.000313**

**p*-values marked in bold are statistically significant (*p*-value < 0.01).*

**FIGURE 2:**
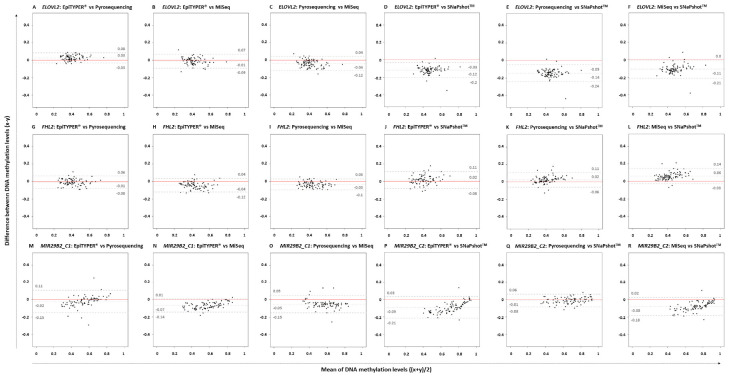
Bland-Altman plots comparing pairs of four DNA methylation technologies: EpiTYPER^®^, pyrosequencing, MiSeq, and SNaPshot^TM^ in *ELOVL2*, *FHL2*, *MIR29B2_C1*, and *MIR29B2_C2* for 84 common controls (18–99 years old). The plots represent the differences between paired methods (*x*–*y*) against the mean of both paired methods [(*x* + *y*)/2] for the following pairs: **(A,G,M)** EpiTYPER^®^ (*x*) vs. pyrosequencing (*y*), **(B,H,N)** EpiTYPER^®^ (*x*) vs. MiSeq (*y*), **(C,I,O)** pyrosequencing (*x*) vs. MiSeq (*y*), **(D,J,P)** EpiTYPER^®^ (*x*) vs. SNaPshot^TM^ (*y*), **(E,K,Q)** pyrosequencing (*x*) vs. SNaPshot^TM^ (*y*), and **(F,L,R)** MiSeq (*x*) vs. SNaPshot^TM^ (*y*). The central dotted gray line represents the mean of the differences; while the discontinuous gray lines represent the upper and lower LoA. The red line indicates the theoretical no differences between methods.

Figure 2 depicts the corresponding paired Bland-Altman plots using the previous DNA methylation values. The central dotted gray line represents the mean of the differences; while the discontinuous gray lines represent the upper and lower LoA, including the 95% of differences between one measurement and the other. The red line indicates the theoretical ‘no differences between methods’. Bland-Altman differences presented a normal distribution for *ELOVL2* and *FHL2* for all pairwise comparisons with the exception of those compared to SNaPshot^TM^ technology (Shapiro–Wilk normality test). Regarding *MIR29B2_C1*, an absence of normality for EpiTYPER^®^ vs. pyrosequencing and for pyrosequencing vs. MiSeq was observed (*p*-value < 0.01). In *MIR29B2_C2*, an absence of normality was found for MiSeq vs. SNaPshot^TM^. Uniformity was found for both *ELOVL2* and *FHL2*, but not for *MIR29B2*. Absence of uniformity was detected for *MIR29B2_C1* for EpiTYPER^®^ vs. pyrosequencing (Figure 2M) and EpiTYPER^®^ vs. MiSeq (Figure 2N), as well as for *MIR29B2_C2* for EpiTYPER^®^ vs. SNaPshot^TM^ (Figure 2P) and for MiSeq vs. SNaPshot^TM^ (Figure 2R) (*p*-value < 0.01). In particular, Figure 2M shows a tendency to overestimate DNA methylation levels with EpiTYPER^®^ vs. pyrosequencing at high values, while DNA methylation levels between 0.5 and 0.2 are underestimated using this technology for *MIR29B2_C1* (Figure 1). Regarding *MIR29B2_C2*, when comparing EpiTYPER^®^ vs. SNaPshot^TM^ (Figure 2P) and for MiSeq vs. SNaPshot^TM^ (Figure 2R), similar DNA methylation values are observed at high values that gradually diverge when DNA methylation values between 0.6 and 0.3 are detected. An additional bias was observed for *MIR29B2_C1* for pyrosequencing vs. MiSeq (Figure 2O), that is explained by an underestimation by pyrosequencing or an overestimation by MiSeq of the DNA methylation levels (Figure 1).

If excluding SNaPshot^TM^ comparisons, the mean of the differences between the DNA methylation levels detected using different technologies for *ELOVL2* and *FHL2* for all pairwise comparisons was quite close to zero (average: ±0.03). SNaPshot^TM^ comparisons for *FHL2* also detected reduced DNA methylation differences between technologies (average: +0.03). However, higher deviations were detected for *ELOVL2* (average: −0.12) due to an overestimation of the DNA methylation levels using SNaPshot^TM^ compared to the other three technologies (Figure 1). This explains a raised value for the lower LoA for *ELOVL2* when including SNaPshot^TM^ analyses (average lower LoA: −0.22) – with values that exceed the established threshold (±0.098). Regarding *MIR29B2* (C1 or C2), for analyses not including SNaPshot^TM^ data, the mean of the differences was reduced (average: −0.04), as the comparison with SNaPshot^TM^ significantly increased these differences (average: −0.085).

### Age Prediction for DNA Methylation Data From EpiTYPER^®^, Pyrosequencing, and MiSeq Using ELOVL2, FHL2, and MIR29B2_C1

**FIGURE 3:**
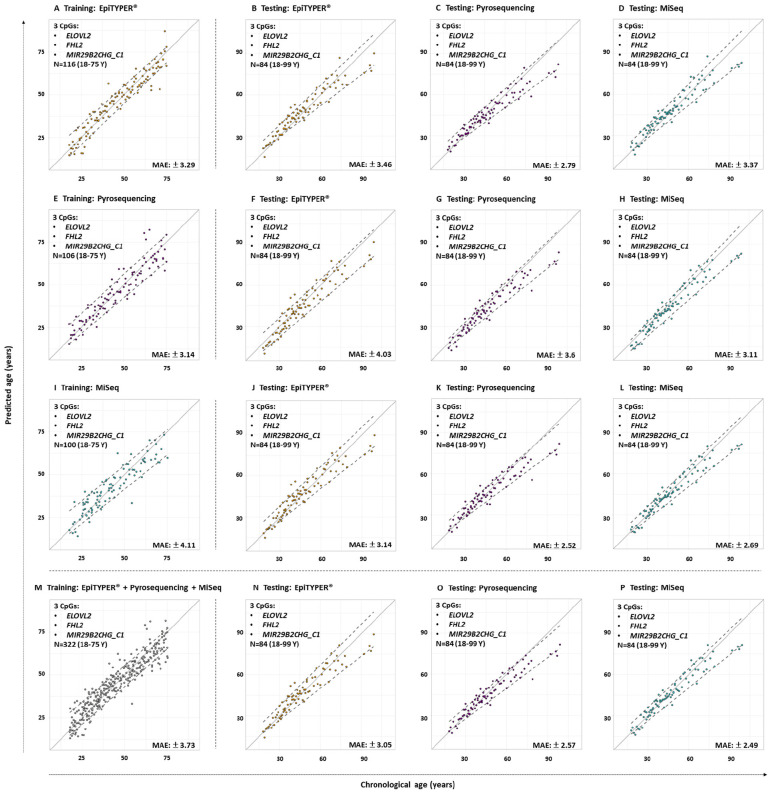
Predicted versus chronological age using four training sets from three DNA methylation technologies using 3-CpG-site models (*ELOVL2*, *FHL2*, and *MIR29B2_C1*) for the 84 common controls (18–99 years old). **(A)** EpiTYPER^®^ training set, **(B–D)** EpiTYPER^®^, pyrosequencing, and MiSeq testing sets analyzed with the EpiTYPER^®^ training set, **(E)** pyrosequencing training set, **(F–H)** EpiTYPER^®^, pyrosequencing, and MiSeq testing sets analyzed with the pyrosequencing training set; **(I)** MiSeq training set, **(J–L)** EpiTYPER^®^, pyrosequencing, and MiSeq testing sets analyzed with the MiSeq training set; **(M)** Combined training set, **(N–P)** EpiTYPER^®^, pyrosequencing, and MiSeq test sets analyzed with the combined training set. The continuous gray line represents perfect correlation. The discontinuous gray lines represent the prediction intervals.

The three CpG sites (*ELOVL2*, *FHL2*, and *MIR29B2_C1*) were used for age estimation of the 84 common controls using the reference training sets based on EpiTYPER^®^, pyrosequencing, and MiSeq. Figure 3 shows the predicted age vs. chronological age for both training and testing sets, and Table 3, the corresponding performance metrics.

**TABLE 3:** Age predictive performance metrics for the training and test sets based on the analysis of three CpG sites (*ELOVL2*, *FHL2*, and *MIR29B2_C1*) using EpiTYPER^®^, pyrosequencing and MiSeq DNA methylation technologies, as well as a combination of all three technologies (Combined).

		**MAE**			
**Technology**	**Group**	**(years)**	**RMSE**	**%CP ± 5**	**%CP ± PI**
**EpiTYPER^®^**	**Training** (*N* = 116)	**±3.29**	**5.15**	**70.53%**	**74.02%**
EpiTYPER^®^	Testing (*N* = 84)	±3.46	5.99	67.07%	70.73%
Pyrosequencing	Testing (*N* = 84)	±2.79	6.48	69.51%	73.17%
MiSeq	Testing (*N* = 84)	±3.37	5.74	69.05%	75%
**Pyrosequencing**	**Training** (*N* = 106)	**±3.14**	**5.9**	**67.55%**	**76.73%**
EpiTYPER^®^	Testing (*N* = 84)	±4.03	6.36	62.2%	78.05%
Pyrosequencing	Testing (*N* = 84)	±3.6	6.79	64.63%	81.71%
MiSeq	Testing (*N* = 84)	±3.11	5.72	72.62%	84.52%
**MiSeq**	**Training** (*N* = 100)	**±4.11**	**6.33**	**57%**	**76%**
EpiTYPER^®^	Testing (*N* = 84)	±3.14	6.08	68.29%	80.49%
Pyrosequencing	Testing (*N* = 84)	±2.52	6.65	69.51%	85.37%
MiSeq	Testing (*N* = 84)	±2.69	5.55	76.19%	85.71%
**Combined**	**Training** (*N* = 322)	**±3.73**	**5.86**	**63.99%**	**77.92%**
EpiTYPER^®^	Testing (*N* = 84)	±3.05	5.93	71.95%	80.49%
Pyrosequencing	Testing (*N* = 84)	±2.57	6.62	74.39%	80.49%
MiSeq	Testing (*N* = 84)	±2.49	5.52	77.38%	84.52%

*MAE: median absolute prediction error, RMSE: root-mean-square error, %CP: percent correct prediction, PI: prediction intervals.*

All four training sets – EpiTYPER^®^, pyrosequencing, MiSeq and the combined training set derived from the combination of the three technologies (Figures 3A,E,I,M, respectively) – provided errors lower than ±5 years (MAE: from ±3.14 to ±4.11) and correct prediction rates higher than 70% (%CP ± PI: from 74.02 to 77.92%). However, for the aim of the present study, an intra-training set comparison rather than an inter-training set comparison was performed, i.e., we compared testing data from the three DNA methylation technologies analyzed using a uniform training set.

From the data modeled using the EpiTYPER^®^ training set, no statistically significant differences were found for the prediction errors shown by the common controls analyzed with EpiTYPER^®^, pyrosequencing or MiSeq (*p*-value > 0.01). However, a general underestimation of age for common controls older than 60 years was detected in pyrosequencing (Figure 3C). Samples analyzed with MiSeq provided the best prediction rates (%CP ± PI: 75%).

The prediction model using the pyrosequencing training set gave no statistically significant differences for the prediction errors in the technologies analyzing common controls (*p*-value > 0.01). In this case, both pyrosequencing and MiSeq underestimated common control age for the whole age range (Figures 3G,H). In spite of this, samples detected with MiSeq provided the best prediction rates (%CP ± PI: 84.52%).

The prediction model using the MiSeq training set gave no statistically significant differences for the prediction errors in the technologies analyzing common controls (*p*-value > 0.01). As with the EpiTYPER^®^ training set, a global underestimation of age for common controls older than 60 years was detected in pyrosequencing (Figure 3K). The best prediction rates were again provided by MiSeq detection (%CP ± PI: 85.71%).

In view of the similarities found for prediction errors and the accurate predictions displayed for common controls, all data from the previous training sets, e.g., EpiTYPER^®^, pyrosequencing, and MiSeq, were combined in order to create a new enlarged platform-independent training set. As before, no statistically significant differences were found for the common control prediction errors in any technology used (*p*-value > 0.01). In common with individual training sets, common controls older than 60 years old were underestimated by pyrosequencing (Figure 3O) and samples detected with MiSeq gave the best prediction rates (%CP ± PI: 84.52%).

### Age Prediction for DNA Methylation Data From EpiTYPER^®^, MiSeq, and SNaPshot^TM^ Using ELOVL2, FHL2, and MIR29B2_C2

**FIGURE 4:**
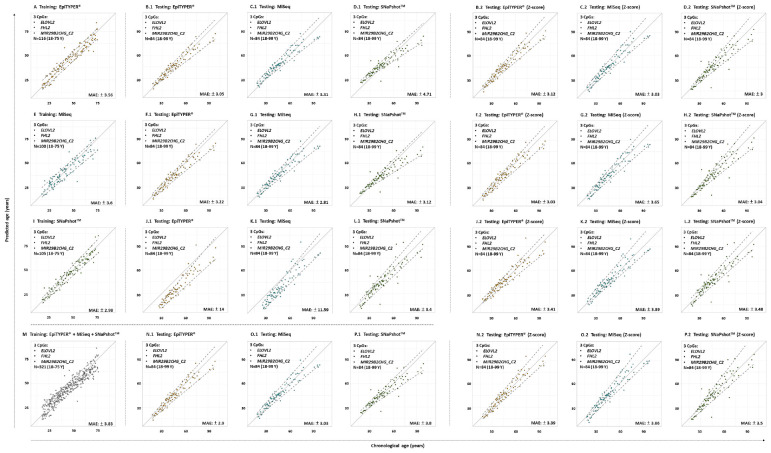
Predicted versus chronological age using four training sets from three DNA methylation technologies and a 3-CpG-site model (*ELOVL2*, *FHL2*, and *MIR29B2_C2*) for the 84 common controls (18–99 years old). **(A)** EpiTYPER^®^ training set, **(B–D)** EpiTYPER^®^, MiSeq, and SNaPshot^TM^ testing sets analyzed with the EpiTYPER^®^ training set, **(E)** MiSeq training set, **(F–H)** EpiTYPER^®^, MiSeq, and SNaPshot^TM^ testing sets analyzed with the MiSeq training set, **(I)** SNaPshot^TM^ training set, **(J–L)** EpiTYPER^®^, MiSeq and SNaPshot^TM^ testing sets analyzed with the SNaPshot^TM^ training set, **(M)** Combined training set, **(N–P)** EpiTYPER^®^, MiSeq and SNaPshot^TM^ testing sets analyzed with the combined training set. Panels **(B.1–P.1)** correspond to untransformed data, while panels **(B.2–P.2)** correspond to z-score transformed data. The continuous gray line represents the perfect correlation. The discontinuous gray lines represent the prediction intervals.

In a subsequent analysis using a different set of CpG sites (*ELOVL2*, *FHL2*, and *MIR29B2_C2*), age prediction was assessed and results plotted in Figure 4, showing the predicted age versus the chronological age using common controls detected with EpiTYPER^®^, MiSeq, and SNaPshot^TM^. Table 4 summarizes the corresponding performance metrics.

**TABLE 4:** Age predictive performance metrics for the training and test sets based on the analysis of three CpG sites (*ELOVL2*, *FHL2*, and *MIR29B2_C2*) using EpiTYPER^®^, MiSeq, and SNaPshot^TM^ DNA methylation technologies, as well as a combination of all three technologies (Combined).

		**MAE**			
**Technology**	**Group**	**(years)**	**RMSE**	**%CP ± 5**	**%CP ± PI**
**EpiTYPER^®^**	**Training** (*N* = 116)	**±3.56**	**4.88**	**72.2%**	**76.59%**
EpiTYPER^®^	Testing (*N* = 84)	±3.05	5.32	71.95%	75.61%
MiSeq	Testing (*N* = 84)	±3.31	5.77	64.29%	58.33%
SNaPshot^TM^	Testing (*N* = 84)	±4.71	7.78	53.57%	44.05%
**MiSeq**	**Training** (*N* = 100)	**±3.6**	**6**	**65%**	**77%**
EpiTYPER^®^	Testing (*N* = 84)	±3.22	5.73	69.51%	74.39%
MiSeq	Testing (*N* = 84)	±2.81	5.83	77.38%	85.71%
SNaPshot^TM^	Testing (*N* = 84)	±3.12	7.96	67.86%	51.19%
**SNaPshot^TM^**	**Training** (*N* = 105)	**±2.98**	**4.43**	**76.09%**	**77%**
EpiTYPER^®^	Testing (*N* = 84)	±14	14.93	4.88%	31.71%
MiSeq	Testing (*N* = 84)	±11.59	12.49	14.29%	47.62%
SNaPshot^TM^	Testing (*N* = 84)	±3.4	8.2	58.33%	60.71%
**Combined**	**Training** (*N* = 321)	**±3.83**	**5.56**	**64.45%**	**79.12%**
EpiTYPER^®^	Testing (*N* = 84)	±2.9	5.27	68.29%	82.93%
MiSeq	Testing (*N* = 84)	±3.03	5.52	73.81%	78.57%
SNaPshot^TM^	Testing (*N* = 84)	±3.8	7.43	63.1%	77.38%

*MAE: median absolute prediction error, RMSE: root-mean-square error, %CP: percent correct prediction, PI: prediction intervals.*

All four training sets – EpiTYPER^®^, MiSeq, SNaPshot^TM^ and the combined training set derived from the combination of the previous three technologies (Figures 4A,E,I,M, respectively) – provided errors lower than ±4 years (MAE: from ±2.98 to ±3.83) and correct prediction rates higher than 75% (%CP ± PI: from 76.59 to 79.12%). Figures 4B.1–4P.1 represent the corresponding testing sets.

For the prediction model using the EpiTYPER^®^ training set, statistically significant differences were found for the prediction errors in the common controls (*p*-value < 0.01). These differences were explained by a higher error rate in SNaPshot^TM^ analysis of common controls (MAE: ±4.71), which was reflected in a decreased correct prediction rate (%CP ± PI: 44.05%). The best predictions were obtained in EpiTYPER^®^ analysis of common controls (%CP ± PI: 75.61%). Although similar errors to those of the initial MiSeq analyses of the training set were found here (MAE: ± 3.31), the correct prediction rate was reduced (%CP ± PI: 58.33%) due to an overestimation of common controls younger than 45 years old (Figure 4C.1).

In spite of not detecting statistically significant differences between the common controls modeled using the MiSeq training set, the correct prediction rate for the SNaPshot^TM^ samples was reduced (%CP ± PI: 51.19%). The best predictions were shown by the MiSeq data (%CP ± PI: 85.71%).

Data modeled using the SNaPshot^TM^ training set presented the highest statistically significant differences (*p*-value = 2e^–16^). Test samples analyzed using either EpiTYPER^®^ or MiSeq displayed high errors (MAE: ±14 and ±11.59, respectively), as well as minimum correct prediction rates (%CP ± PI: 31.71 and 47.62%, respectively).

In spite of these differences, a model using all the previous training sets was combined into a single platform-independent training set (Figure 4M). This combined model harmonized the data derived from all the technologies where prediction errors had no statistically significant differences (*p*-value > 0.01). Accordingly, similar correct prediction rates were obtained for all common controls detected using EpiTYPER^®^, MiSeq, and SNaPshot^TM^ (82.93, 78.57, and 77.38%, respectively).

Due to the differences encountered for SNaPshot^TM^ analyses when compared to EpiTYPER^®^ and MiSeq, a z-score transformation was applied in order to check if the corresponding predictions could be improved by data scaling (Table 5). The application of a z-score transformation removed the previously encountered statistical differences. The EpiTYPER^®^ and MiSeq test sets were markedly improved when modeled with the SNaPshot^TM^ training set (Figures 4J.2,K.2, %CP ± PI: 78.05 and 75% in comparison to the previous 31.71 and 47.62%, respectively). Similarly, the SNaPshot^TM^ test set substantially improved when modeled with EpiTYPER^®^ and MiSeq training sets (Figures 4D.2,H.2, %CP ± PI: 77.38 and 85.71% in comparison to previous values of 44.05 and 51.19%, respectively).

**TABLE 5:** Age predictive performance metrics based on a z-score transformation for the training and test sets analyzing three CpG sites (*ELOVL2*, *FHL2*, and *MIR29B2_C2*) and using EpiTYPER^®^, MiSeq, and SNaPshot^TM^ DNA methylation technologies, plus the combination of all three technologies (Combined).

		**MAE**			
**Technology**	**Group**	**(years)**	**RMSE**	**%CP ± 5**	**%CP ± PI**
**EpiTYPER^®^**	**Training** (*N* = 116)	**±3.56**	**4.88**	**72.2%**	**76.59%**
EpiTYPER^®^	Testing (*N* = 84)	±3.12	5.08	75.61%	80.49%
MiSeq	Testing (*N* = 84)	±3.03	5.41	75%	80.95%
SNaPshot^TM^	Testing (*N* = 84)	±3	7.09	70.24%	77.38%
**MiSeq**	**Training** (*N* = 100)	**±3.6**	**6**	**65%**	**77%**
EpiTYPER^®^	Testing (*N* = 84)	±3.03	5.4	70.24%	90.24%
MiSeq	Testing (*N* = 84)	±3.65	5.75	63.1%	88.1%
SNaPshot^TM^	Testing (*N* = 84)	±3.04	7.35	76.09%	85.71%
**SNaPshot^TM^**	**Training** (*N* = 105)	**±2.98**	**4.43**	**76.09%**	**77%**
EpiTYPER^®^	Testing (*N* = 84)	±3.41	6.15	70.73%	78.05%
MiSeq	Testing (*N* = 84)	±3.89	6.61	66.67%	75%
SNaPshot^TM^	Testing (*N* = 84)	±3.48	7.81	71.43%	77.38%
**Combined**	**Training** (*N* = 321)	**±3.83**	**5.56**	**64.45%**	**79.12%**
EpiTYPER^®^	Testing (*N* = 84)	±3.39	5.49	69.51%	85.37%
MiSeq	Testing (*N* = 84)	±3.66	6.09	64.29%	80.95%
SNaPshot^TM^	Testing (*N* = 84)	±3.5	7.67	65.48%	82.14%

*MAE: median absolute prediction error, RMSE: root-mean-square error, %CP: percent correct prediction, PI: prediction intervals.*

## Discussion

Correlation has been proposed as a statistical technique in order to compare technologies (Hong et al., 2019). However, this parameter evaluates the relationship or association between one variable and another, not their differences. In order to compare the differences between two measurement methods in our study, Bland-Altman analysis was applied (Giavarina, 2015). Bland-Altman analysis describes the degree of agreement between two quantitative technologies for the same variable (Bland and Altman, 1999) – in our case, between four DNA methylation detection methods. With this analysis, 95% of the differences between two methods are plotted within the LoA. It is important to consider that the maximum accepted LoA should be established before the analysis, according to analytical or biological criteria. Since some intra-technical variance was already accepted for DNA methylation (SD ≤ 0.05 for replicates) (Freire-Aradas et al., 2016), inter-technical deviation based on the Bland-Altman’s LoA and previous intra-technical variance was explored in the present study (±1.96 SD = ±0.098). In addition to the LoA, it is also recommended that the differences between technologies are normally distributed, although not essential. However, uniformity is required before data can be used interchangeably.

Four DNA methylation technologies were compared using four CpG sites from *ELOVL2*, *FHL2*, and *MIR29B2*. Normality was analyzed for all the pairwise comparisons. Differences were normally distributed for *ELOVL2* and *FHL2* for all pairwise comparisons, except for *ELOVL2* in EpiTYPER^®^/pyrosequencing/MiSeq vs. SNaPshot^TM^, and *MIR29B2* presented partial normal distribution. However, absence of normality can be handled in our study since subjects were not chosen randomly, but to give a wide distribution of the factor measured. The critical parameter that is required to exchange data among technologies without affecting the outcome is uniformity (Bland and Altman, 1999). Uniformity can be observed as the absence of a tendency for the differences to change between methods, i.e., the extent of the differences is uniformly maintained independently of the magnitude of the variable. In our study, uniformity is measured as the variance in the differences across the range of DNA methylation levels, and this should be maintained. The *ELOVL2* and *FHL2* CpG sites in the present work displayed complete uniformity for all comparisons made. The same cannot be said for *MIR29B2*. This marker had an evident tendency to show changes in differences in several comparisons (Figures 2M,N,P,R; *p*-value < 0.1). Some of them are explained by similar DNA methylation detection at high DNA methylation levels that gradually diverge – increasing the value of the differences when DNA methylation levels between 0.6 and 0.3 are detected (Figure 1, *MIR29B2_C2*). However, *MIR29B2_C1* for MiSeq vs. pyrosequencing behaves in the opposite way; i.e., bigger differences are found at high DNA methylation levels, that progressively decrease when DNA methylation values at about 0.3 are detected (Figure 1).

With regard to the DNA methylation levels, the highest levels of similarity were displayed by the quantitative technologies of EpiTYPER^®^, pyrosequencing and MiSeq, especially for *ELOVL2* and *FHL2*. Nevertheless, some differences among these technologies were found in *MIR29B2*, although not statistically significant (*p*-value > 0.01) (Figures 2M–R) as they slightly exceeded the LoA from the established threshold of ±0.098 (average lower LoA: −0.15). However, it can be concluded from our findings that when differences are uniformly within an established LoA, then methodologies can be used interchangeably (Bland and Altman, 1999). In order to test if the differences observed in the DNA methylation values are critical or not, the corresponding age predictions were performed using four re-configured age prediction models constructed using DNA methylation data detected with EpiTYPER^®^ (Freire-Aradas et al., 2016), pyrosequencing (Zbieć-Piekarska et al., 2015), and MiSeq (Aliferi et al., 2018). In this way, despite differences encountered in *MIR29B2*, the prediction accuracy of the corresponding age prediction models when comparing EpiTYPER^®^, pyrosequencing, and MiSeq was not affected (Figure 3 and Table 3). Nevertheless, it is important to note the underestimation of predicted age using pyrosequencing (Figures 3C,G,K,O) to test common controls older than 60 years.

In addition to this, previous replication experiments had indicated reproducibility for DNA methylation levels detected using EpiTYPER^®^ (Bocklandt et al., 2011), MPS (Pabinger et al., 2016), and pyrosequencing (Bocklandt et al., 2011) compared with data from Infinium BeadChip arrays. This is an important factor to consider, since discovery studies are predominantly based on Infinium arrays, and identified age-associated CpG sites are subsequently validated using targeted DNA methylation technologies.

Different patterns are obtained when SNaPshot^TM^ analyses are included. SNaPshot^TM^ is a semi-quantitative method and this is reflected in the differences detected for estimated methylation levels. Due to a lack of overlap among the *MIR29B2* CpG sites between technologies, an independent comparison was performed in order to include SNaPshot^TM^ analyses. The major differences were found for *ELOVL2* and *MIR29B2_C2* when comparing either EpiTYPER^®^ or MiSeq with SNaPshot^TM^ (Figures 2D–F,P–R). The main difference between both markers is the lack of uniformity for *MIR29B2_C2*, with a tendency to generate more differences when detecting DNA methylation levels between 0.6 and 0.3 (Figure 1). However, differences for *ELOVL2*, although present, are almost proportional between methods (Figure 1). On the other hand, SNaPshot^TM^ analysis of *FHL2* provided more similarities when compared with EpiTYPER^®^ or MiSeq (mean of the differences: +0.02 and +0.06, respectively). Differences encountered for SNaPshot^TM^ could be explained by differences in the intensity of the fluorochromes; as previously reported by Hong et al. (2019). In spite of the differences in *ELOVL2* and *MIR29B2_C2*; it is important to note that all three markers, including *FHL2*, were genotyped using CT dyes and detected using an ABI3130. The CT dyes used in SNaPshot^TM^ are characterized by more closely matched intensities in terms of fluorescence, and theoretically should provide unbiased DNA methylation values, more similar to those obtained using quantitative technologies than sites detected with the AG SNaPshot^TM^ dyes. Our results agree this assumption only for *FHL2*, so additional factors are likely to be affecting the results for *ELOVL2* and *MIR29B2_C2*. The effect of such differences is reflected in the age prediction accuracies (Table 4). Either using the EpiTYPER^®^ or MiSeq training set, with the worst predictions obtained for data analyzed using SNaPshot^TM^. In view of these results, SNaPshot^TM^ data cannot be used with prediction models based on EpiTYPER^®^ or MiSeq technology. However, it has been observed that if expanding the training set to data from the three technologies, i.e., EpiTYPER^®^, MiSeq, and SNaPshot^TM^ (combined training set), SNaPshot^TM^ common controls are correctly predicted at a similar rate to those detected with EpiTYPER^®^ or MiSeq.

Although the training sets used in the prediction models from the different DNA methylation technologies were harmonized in terms of sample size, age distribution and the underlying statistical model, factors potentially affecting technical variation such as bisulfite conversion, DNA input, amplicon length or PCR cycles should be taken into account (Supplementary Table S1). One of the main factors affecting methylation results is the efficiency of bisulfite conversion. The acid pH and high temperatures accompanying this molecular process lead to DNA fragmentation. It has been observed that different DNA degradation rates can be encountered if using different bisulfite conversion kits (Kint et al., 2018). Since fragmentation usually leads to sequences smaller than 500 bp (Grunau et al., 2001), a reduced amplification of longer amplicons could occur, although the exact effects remain unknown. Differences in DNA input could also affect results, although this should be linked to the manufacturer’s recommendations as well as to the levels of technical optimization achieved for each methodology. Variation in DNA methylation levels could also be explained by biological variation. Although in order to minimize this effect, common test samples were represented by a single individual per year (18–99 years old), biological variation cannot be discounted since differences in white blood cell composition could alter DNA methylation levels.

## Concluding Remarks

To the best of our knowledge, this is the first study covering the broadest possible comparison between DNA methylation technologies currently applied to forensic age prediction. Interchangeability of methylation data was found to be a suitable strategy when differences in the DNA methylation levels from different technologies do not exceed the uniformity threshold established by this study of ±0.098 (±1.96.SD, SD = 0.05), and maintain this uniformity across the range of DNA methylation values detected. If the differences slightly exceed the threshold, it should be confirmed that these variations are not relevant for age estimation. Although the CpG sites for *ELOVL2*, *FHL2*, and *MIR292B* covered by the present study provide high accuracy for age prediction for most of the comparisons performed, in *MIR292B* the LoA is exceeded, and a lack of uniformity is consistently observed. Therefore, DNA methylation data for *MIR292B* should not be used independently of the technology applied. These deviations could be explained by internal technical problems, which we have observed for this gene (additional methylation studies with publications in preparation). In *ELOVL2* and *FHL2*, similar patterns of DNA methylation for EpiTYPER^®^, pyrosequencing, and MiSeq were observed, and subsequently data from these techniques can be used in platform-independent age prediction models. However, our results are linked to specific CpG positions – so no general extrapolations can be assumed. If additional CpG sites from *ELOVL2* and *FHL2* are considered for inclusion in technology-free age prediction models, the necessary validation tests should be made.

SNaPshot^TM^ is a semi-quantitative technology based on fluorescence using dyes with different signal intensities. This introduces a bias in the DNA methylation values detected that explains the differences found for *ELOVL2* and *MIR292B_C2* in SNaPshot^TM^ compared with EpiTYPER^®^ or MiSeq, which subsequently decrease the accuracy of corresponding age predictions.

If differences are encountered between technologies; two viable corrective measures could be applied, as proposed by previous studies: (a) a z-score transformation in order to solve batch effects (Feng et al., 2018) or; (b) the addition of an extra covariate in the model indicating the type of technology used, then introducing a correction for the method (Hong et al., 2019). When a z-score transformation was tested in the present study it markedly improved the results when SNaPshot^TM^ data was included in the analyses. If applying a platform-independent model, there is a risk of losing age prediction accuracy if the underlying sample size does not match the sample size of the original age prediction model. Further work to increase the number of samples tested among technologies will be necessary to detect if prediction accuracies are affected by different sample sizes. On the other hand, a re-analysis of the corresponding training set using the technology of interest would be also be a viable approach.

## Data Availability Statement

All datasets presented in this study are included in the article/Supplementary Material.

## Ethics Statement

Ethical approval was granted from the Ethics Committee of investigation in Galicia, Spain (CAEI: 2013/543).

## Author Contributions

AF-A, CP, EP, AA, WB, DB, DC, ÁC, and ML devised the experiments. AF-A, EP, AA, LG-S, AM-M, AP, AA-C, MS, and AW performed the laboratory work. AF-A, EP, AA, MC, AG-T, JÁ-D, DB, and WB compiled and analyzed the data. AF-A and CP wrote the manuscript. All authors contributed to the article and approved the submitted version.

## Conflict of Interest

The authors declare that the research was conducted in the absence of any commercial or financial relationships that could be construed as a potential conflict of interest.

## Supplementary Material

FIGURE S1 DNA methylation levels against the chronological age for *ELOVL2*, *FHL2*, and *MIR29B2_C2* for both SNaPshot^TM^ replicates for the 84 common controls (18–99 years old).

TABLE S1 Summary of variable factors between DNA methylation technologies.

TABLE S2 Specific multiplex protocol details for SNaPshot^TM^.

TABLE S3 DNA methylation data (β-values) for *ELOVL2*, *FHL2*, *MIR29B2_C1*, and *MIR29B2_C2* for the 84 common controls (18–99 years old) analyzed using EpiTYPER^®^, pyrosequencing, MiSeq, and SNaPshot^TM^.
