# Supplementary figures and images for: A Comparison of Forensic Age Prediction Models Using Data From Four DNA Methylation Technologies
Source: Front Genet. 2020 Aug 19;11:932. doi: 10.3389/fgene.2020.00932 (PMC7466768; doi:10.3389/fgene.2020.00932)

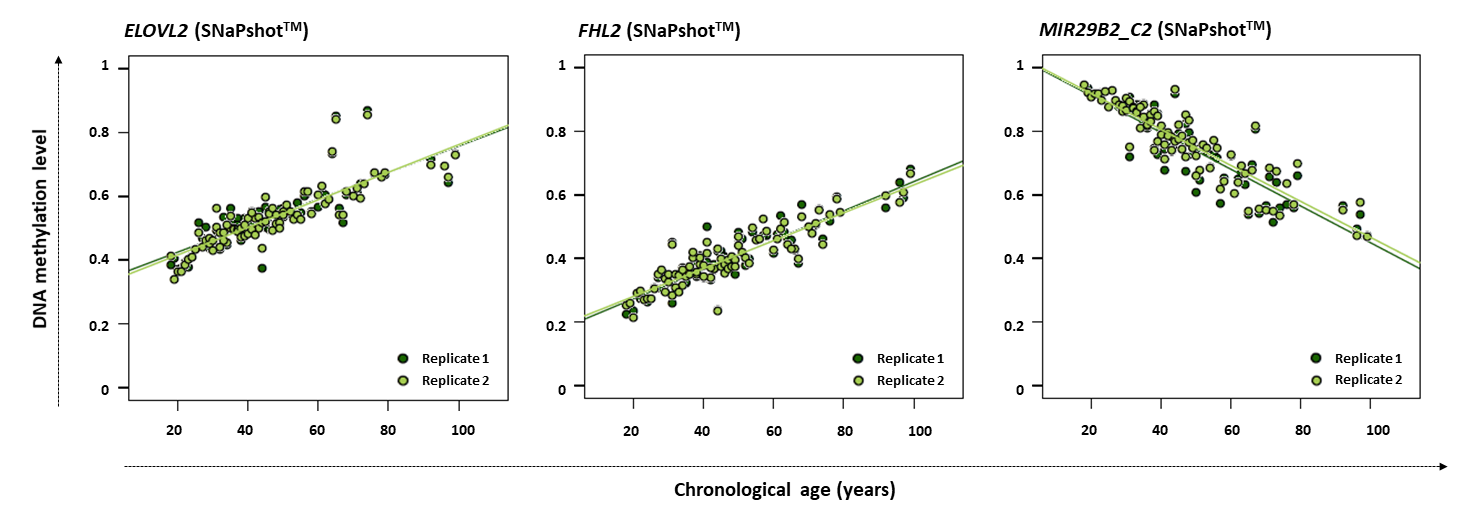

Supplement: FIGURE S1 — DNA methylation levels against the chronological age for ELOVL2, FHL2, and MIR29B2_C2 for both SNaPshotTM replicates for the 84 common controls (18–99 years old). [file Image_1.TIF]
